# Tailoring and Evaluating Treatment with the Patient-Specific Needs Evaluation: A Patient-Centered Approach

**DOI:** 10.1097/PRS.0000000000011199

**Published:** 2023-12-12

**Authors:** Willemijn A. de Ridder, Yara E. van Kooij, Harm P. Slijper, Grada R. Arends, Aaltje de Roode, Joy C. MacDermid, Guus M. Vermeulen, Steven E.R. Hovius, Ruud W. Selles, Robbert M. Wouters

**Affiliations:** Rotterdam, Amsterdam, Utrecht, and Nijmegen, the Netherlands; and London, Ontario, Canada; From the Departments of 1 Plastic, Reconstructive, and Hand Surgery; 2 Rehabilitation Medicine, Erasmus Medical Center, University Medical Center Rotterdam; 3 Hand and Wrist Center, Xpert Clinics; 4 Center for Hand Therapy, Xpert Handtherapie; 5 Department of Plastic, Reconstructive, and Hand Surgery, Radboud University Medical Center University Hospital; 6 School of Physical Therapy, Department of Surgery, Western University; 7 Fysio- & Oefentherapie Rhenen-Elst.

## Abstract

**Background:**

No patient-reported instrument assesses patient-specific information needs, treatment goals, and personal meaningful gain (PMG), a novel construct evaluating individualized, clinically relevant improvement. This study reports the development of the Patient-Specific Needs Evaluation (PSN) and examines its discriminative validity (ie, its ability to distinguish satisfied from dissatisfied patients) and test-retest reliability in patients with hand or wrist conditions.

**Methods:**

A mixed-methods approach was used to develop and validate the PSN, following Consensus-Based Standards for the Selection of Health Measurement Instruments guidelines, including pilot testing, a survey (pilot, *n* = 223; final PSN, *n* = 275), cognitive debriefing (*n* = 16), expert input, and validation. Discriminative validity was assessed by comparing the satisfaction level of patients who did and did not achieve their PMG (*n* = 1985) and test-retest reliability using absolute agreement, the Cohen kappa, and intraclass correlation coefficients (*n* = 102). The authors used a sample of 2860 patients to describe responses to the final PSN.

**Results:**

The PSN has only 5 questions (completion time, ±3 minutes) and is freely accessible online. The items and response options were considered understandable by 90% to 92% of the end-users and complete by 84% to 89%. The PSN had excellent discriminative validity (Cramer V, 0.48; *P* < 0.001) and moderate to high test-retest reliability (kappa, 0.46 to 0.68; intraclass correlation coefficients, 0.53 to 0.73).

**Conclusions:**

The PSN is a freely available, patient-centered decision support tool that helps clinicians tailor their consultations to patients’ individual needs and goals. It contains the PMG, a novel construct evaluating individualized, clinically relevant treatment outcomes. The PSN may function as a conversation starter, facilitate expectation management, and aid shared decision-making. The PSN is implementation-ready and can be readily adapted to other patient populations.

Patient-centered and value-based health care frameworks have gained global recognition in recent years, with the aims of putting the patient first and achieving better outcomes at lower costs.^1–4^ Key in these frameworks is responding to individual information needs and treatment goals,^5^ aiming for high satisfaction with the treatment results.^6–11^ It is important, therefore, for clinicians to be well informed about the patient’s information needs and treatment goals. Clinicians aim to meet patients’ needs and goals, but sometimes a misalignment occurs. For instance, a surgeon may prioritize pain relief with a joint replacement, while the patient prioritizes hand appearance. This misalignment can induce a treatment plan that does not fully meet the patient’s needs or goals.

In routine care, clinicians must understand each patient’s information needs, as patients require information to comprehend their medical situation, participate in decision-making, and manage expectations. Providing targeted, patient-specific information improves shared decision-making,^12^ daily functioning,^13^ treatment adherence,^14^ quality of life, the patient’s mindset, pretreatment expectations,^15–23^ and satisfaction with care and treatment results.^24^ Since information provision is modifiable,^25–28^ outcomes can be improved. There is a lack of concise, patient-reported tools that focus on patients’ information needs and treatment goals. These needs and goals may be, for example, understanding the diagnosis or regaining the ability to perform daily activities. Setting goals enhances patient participation, treatment adherence, and motivation, thereby ultimately improving outcomes and satisfaction with treatment results.^29–31^ There are several limitations to existing tools that assess patient-specific limitations or goals, including the Canadian Occupational Performance Measure,^32^ Goal Attainment Scaling,^33^ the Patient-Specific Goalsetting Method,^34^ and the Patient-Specific Functional Scale.^35^ These limitations depend on the specific tool and include being time-consuming,^31^ having the potential for therapist bias,^32–34^ and only focusing on the activities and participation levels instead of all levels of the International Classification of Functioning, Disability, and Health.^32–36^ Moreover, they do not assess patient-specific improvement goals (ie, when is the patient satisfied with the treatment results?). Patient-specific improvement goals may depend on condition, treatment type, baseline score, and other patient-specific factors. For example, a recreational tennis player may consider a change from 4 to 8 on a scale of 0 to 10 to be satisfactory, whereas a professional tennis player may not. We introduce the personal meaningful gain (PMG) to represent the improvement an individual wants to obtain in a domain relevant to that individual, given the baseline score. Knowing the patient’s information needs, individual goal, and PMG before treatment will allow clinicians to improve decision support and facilitate expectation management.

This study introduces the Patient-Specific Needs Evaluation (PSN), a brief patient-reported tool that assesses patient-specific information needs, treatment goals, and PMG before a first clinician consultation. Specifically, the first objective of this study was to describe the development of the brief, easy-to-use patient-reported tool to assess patient-specific information needs, treatment goals, and PMG. This tool was initially developed for patients with hand and wrist conditions, but we designed it to be easily adapted in other patient populations. The second study objective was to examine the PSN’s discriminative validity (ie, its ability to distinguish satisfied from dissatisfied patients) and test-retest reliability. The third study objective was to describe the results of the final PSN.

## METHODS

### Study Design

This was a user-centered mixed-methods study of patients with hand or wrist conditions, health care providers, and other stakeholders. We used the Consensus-Based Standards for the Selection of Health Measurement Instruments guidelines on patient-reported outcome measure development and measurement properties.^37^

### Setting

We developed the PSN at Erasmus Medical Center (an academic hospital) and Xpert Clinics (a specialized clinic for hand and wrist care) in the Netherlands. Data were collected at Xpert Clinics^20^ between July and August of 2023. The medical ethics review committee of Erasmus Medical Center approved this study, and all participants provided informed consent.

### Research Team

The core research team consisted of hand surgeons and therapists (W.A.d.R., Y.E.v.K., R.M.W., S.E.R.H., G.R.A., A.d.R., G.M.V., and J.C.M.), professionals with experience in developing measurement sets and tools (R.M.W., S.E.R.H., H.P.S., J.C.M., and R.W.S.),^11,38–41^ and electronic data capturing and implementation experts (H.P.S., Y.E.v.K., R.M.W., R.W.S., S.E.R.H., J.C.M., G.M.V., and W.A.d.R.).^20,42^ We consulted other clinicians, language experts, and native English speakers.

### PSN Development Process

**Fig. 1.**
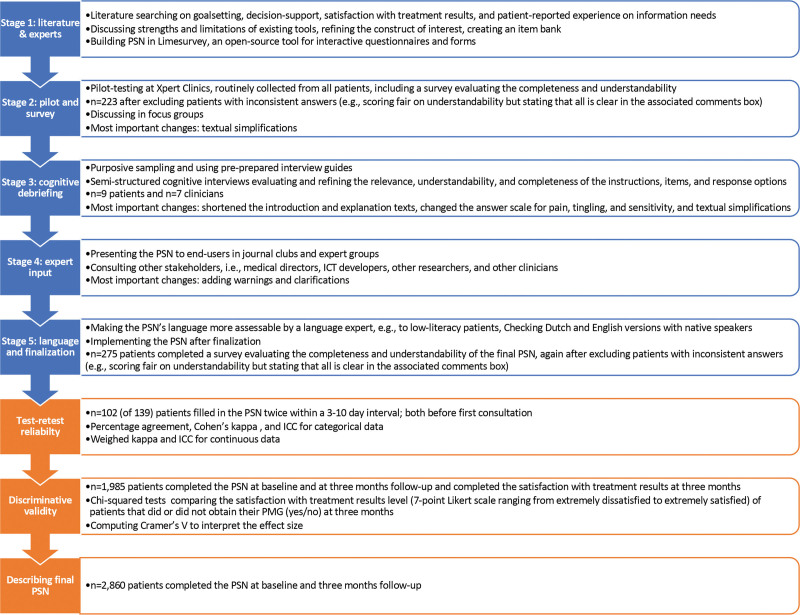
Flow chart of the development (in *blue*) and validation (in *orange*) of the PSN, describing the sample and the most important goals and activities per stage.

Development of the PSN was iterative and comprised 5 overlapping stages, with each stage informing subsequent stages (Fig. 1). Stage 1 included literature studies and expert meetings. After developing an item bank, we conducted a pilot study and survey on completeness and understandability in stage 2. Stage 3 included cognitive debriefing of patients and clinicians and refining of the item bank. We gathered expert input in stage 4, and consulted a language expert, performed crosscultural translation, and repeated the survey for the final PSN in stage 5 (for more details, see Fig. 1).

### Participants

We used different samples to develop the PSN and establish the discriminative validity and test-retest reliability (Fig. 1). For all samples, patients were eligible if they were adults, had any hand or wrist condition, completed our intake questionnaire, and understood the Dutch language. All questionnaires were completed digitally.

For the survey, we excluded patients who gave inconsistent answers (eg, stating “fair” on understandability but stating that all is clear in the associated comments box).

For discriminative validity, we included patients who completed the PSN at baseline and at 3-month follow-up, as well as the Satisfaction with Treatment Results Questionnaire at 3 months.^10,11^ We prospectively invited patients to participate in a test-retest study and complete the PSN for a second time 3 to 5 days after initial completion. The retest remained accessible for 6 days (ie, a possible time interval of 3 to 11 days). We hypothesized that patient needs and goals would remain stable during this period. We included patients in the test-retest analysis if they completed both the primary and retest PSN before clinician consultation. The Consensus-Based Standards for the Selection of Health Measurement Instruments advise a sample size of more than 100 participants when examining test-retest reliability.^43^ To describe the results of the final PSN, we included all patients who completed the PSN at baseline and at 3-month follow-up. There were no additional exclusion criteria. All samples reflected the target population (patients with hand and wrist conditions) and differed in age, sex, and treatment location.

### Discriminative Validity, Test-Retest Reliability, and Statistical Analysis

We evaluated discriminative validity by comparing the satisfaction with treatment results level of patients who did and did not obtain their PMG. At 3 months, we used a Satisfaction with Treatment Results questionnaire,^10,11^ which evaluates satisfaction using a 7-point Likert scale ranging from extremely dissatisfied to extremely satisfied. Using chi-squared tests, we determined the PMG’s discriminative power. We computed the Cramer V to interpret the effect size, where 0.10 reflects a small effect size, 0.30 reflects a medium effect size, and 0.50 reflects a large effect size.^44^

We evaluated test-retest reliability by computing the absolute agreement and Cohen kappa. We computed intraclass correlation coefficients for all variables, including goal domain, baseline score, score needed to be satisfied with the most important goal domain, and PMG. Kappa scores lie between −1 and 1, with 0 or less indicating no agreement; 0.01 to 0.20, no to slight agreement; 0.21 to 0.40, fair agreement; 0.41 to 0.60, moderate agreement; 0.61 to 0.80, substantial agreement; and 0.81 to 1.00, almost perfect agreement.^45^ We calculated intraclass correlation coefficients using a 2-way mixed-effects model.^46^ The intraclass correlation coefficients range from 0 to 1, with 1 being perfect reliability; 0.90 to 0.99, very high reliability; 0.70 to 0.89, high reliability; 0.50 to 0.69, moderate reliability; 0.26 to 0.49, low reliability; and 0.00 to 0.25, little, if any, reliability.^47–49^

There were no missing data in the final PSN, as completing it before clinician consultation is mandatory in our clinical setting. We analyzed missing data patterns for the test-retest analyses. Patients who completed both the primary and retest tests were responders, and patients without a retest were nonresponders. We compared baseline characteristics of responders and nonresponders using significance testing and calculating standardized mean differences to investigate whether they differed systematically. R statistical software version 4.1.1 was used for the quantitative analyses, and *P* < 0.05 was considered significant. We tested the Dutch version of the PSN.

## RESULTS

### Development Process: Cognitive Debriefing and Survey Data

We performed 16 cognitive interviews among 9 patients and 7 clinicians. All patients (3 men and 6 women; age range, 21 to 71 years; median age, 51 years) had different diagnoses. We also included patients with lower levels of education. Among clinicians, we interviewed 1 occupational hand therapist, 2 physical hand therapists, and 4 hand surgeons (5 men and 2 women, age range, 27 to 70 years; median age, 40 years). We iteratively improved the PSN, alternating between interviewing and adjusting (eg, we shortened the introduction and explanation texts; changed the answer scale for pain, tingling, and sensitivity; and simplified the text with a language expert). (**See Table, Supplemental Digital Content 1**, which shows the conceptual framework of the PSN derived from cognitive interviews with patients [*n* = 9], http://links.lww.com/PRS/H13. **See Table, Supplemental Digital Content 2**, which shows the conceptual framework of the PSN derived from cognitive interviews with clinicians [*n* = 7], http://links.lww.com/PRS/H14.)

The survey on the final PSN indicated that the questions and answer options were rated entirely or mostly understandable by 90% to 92% and fully or mostly complete by 84% to 89% of the 275 participants. (**See Figure, Supplemental Digital Content 3**, which shows pie charts indicating the understandability and completeness of the questions and response options on information needs [*A, B*, and *C*], treatment goals, and PMG [*D, E*, and *F*]. The survey indicated that 90% considered the questions on information need entirely or mostly understandable, 91% considered the answer options entirely or mostly understandable, and 84% rated the answer options as entirely or mostly complete. For the treatment goals and PMG, this was 92%, 91%, and 89%, respectively, http://links.lww.com/PRS/H15.) For the pilot PSN (*n* = 223), the questions and answer options were rated entirely or mostly understandable by 89% to 93% and fully or mostly complete by 86% to 91%.

### The Final PSN

**Fig. 2.**
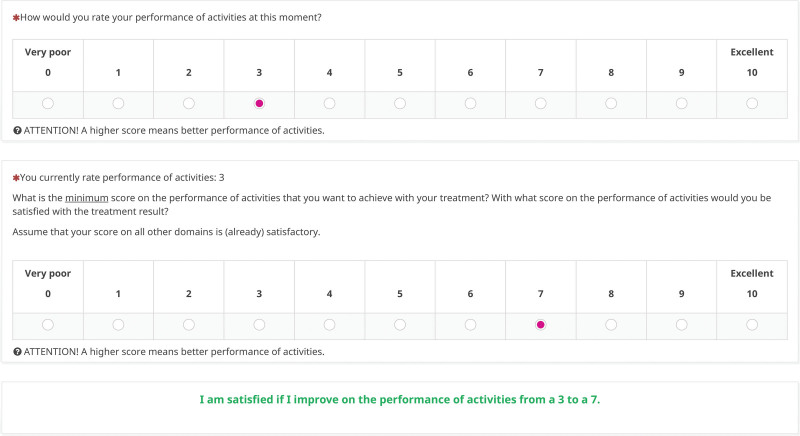
Visual of the PSN treatment goal and PMG. In this example, the patient entered that the most important treatment goal was to improve the performance of activities. The score at baseline was 3 on a scale of 0 to 10 (high scores indicate better performance), and the patient indicated that a score of 7 is needed to become satisfied with the treatment result. After this section is filled in, the digital PSN automatically generates a statement on the treatment goal and the PMG, so the patient can check whether it is correct or needs modification.

Because of the dependencies within the PSN, it works best in digital form. It can be accessed at https://personeel.equipezorgbedrijven.nl/ls/index.php?r=survey/index&sid=587344&lang=en (see Table 1 for a nondigital version). The intake PSN has 5 questions and takes approximately 3 minutes to complete. The information needs section asks an open question about the patient’s reason for making an appointment at the clinic (the patient’s request for help), followed by a single-select question where respondents pick their most important information need category. Respondents then select a predefined subanswer based on that category, to specify their information need in more detail. The treatment goal section of the PSN asks respondents to choose which domain they would most like to improve if they were to be treated and to rate their baseline score on that domain on a scale of 0 to 10 (eg, the baseline pain score). Respondents have the option of selecting 2 secondary goal domains. The final question asks for the score they think they need to achieve with treatment to be satisfied. The PMG is then generated automatically as the difference between the respondent’s baseline performance rating and the score needed for the patient to be satisfied (Fig. 2). The follow-up PSN evaluates the previously selected information needs and treatment and improvement goals in only 2 questions, and takes less than 1 minute to complete.

**Table 1.** The Nondigital Version of the PSN Questionnaire^a^

Section	Question	Response Options
Information needs	1. What is the reason that you have made an appointment with us? In other words, what is your request for help from the doctor?	[Open text]
	2A. What is your most important information need?	Choose one of the following options: • I do not need information • Diagnosis (I have questions about the diagnosis) • Advice (I want to know what is the best thing to do in my situation) • Treatment (I have questions about the treatment) • Perspective (I want to know what to expect in the future)
	2B. Specifying question based on information need: • On which topic would you like advice? OR • What would you like to know about the diagnosis? OR • What would you like to know about the treatment? OR • What would you like to know about your perspective?	[Choose one of the response options depending on information need category; see digital PSN for all options]
Treatment and improvement goals	3. If you were treated, which domain would you most like to improve?	Choose one of the following options: • I do not want to be treated • Numbness (loss of sensation) • Mobility/flexibility of my hand • Strength • Pain • Tingling • Performance of activities (eg, housekeeping, hobby, sports) • Appearance of my hand/wrist • Ability to work
	4. How would you rate your [domain from question 3] at this moment?	Score range 0 to 10; higher scores indicate better performance, except for the items “numbness (loss of sensation),” “pain,” and “tingling”
	5. What is the minimum score on [domain] that you want to achieve with your treatment? With what score would you be satisfied with the treatment result? Assume that your score on all other domains is (already) satisfactory.	Score range 0 to 10; higher scores indicate better performance, except for the items “numbness (loss of sensation),” “pain,” and “tingling”

a The PSN is best administered in digital form and can be accessed digitally via open access at https://personeel.equipezorgbedrijven.nl/ls/index.php?r=survey/index&sid=587344&lang=en. Table 1 displays each question and the associated response options, which, in some specific domains, are slightly different from those displayed. After question 4, respondents have the option to pick 2 secondary domains.

**Fig. 3.**
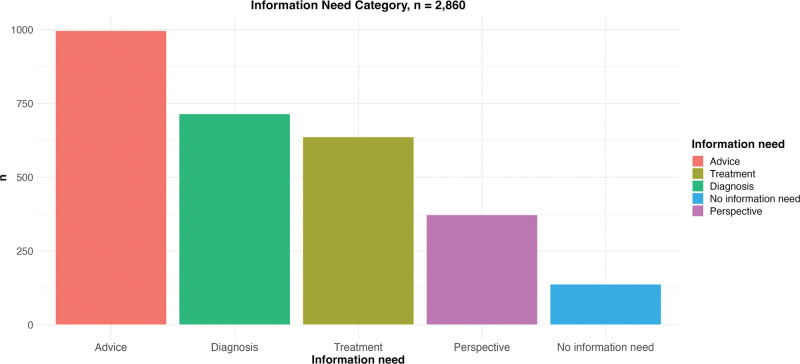
Distribution of information need in the final version of the PSN. The patient chooses one of the following options: I do not need information; diagnosis (I have questions about the diagnosis); advice (I want to know what is the best thing to do in my situation); treatment (I have questions about the treatment); or perspective (I want to know what to expect in the future).

**Fig. 4.**
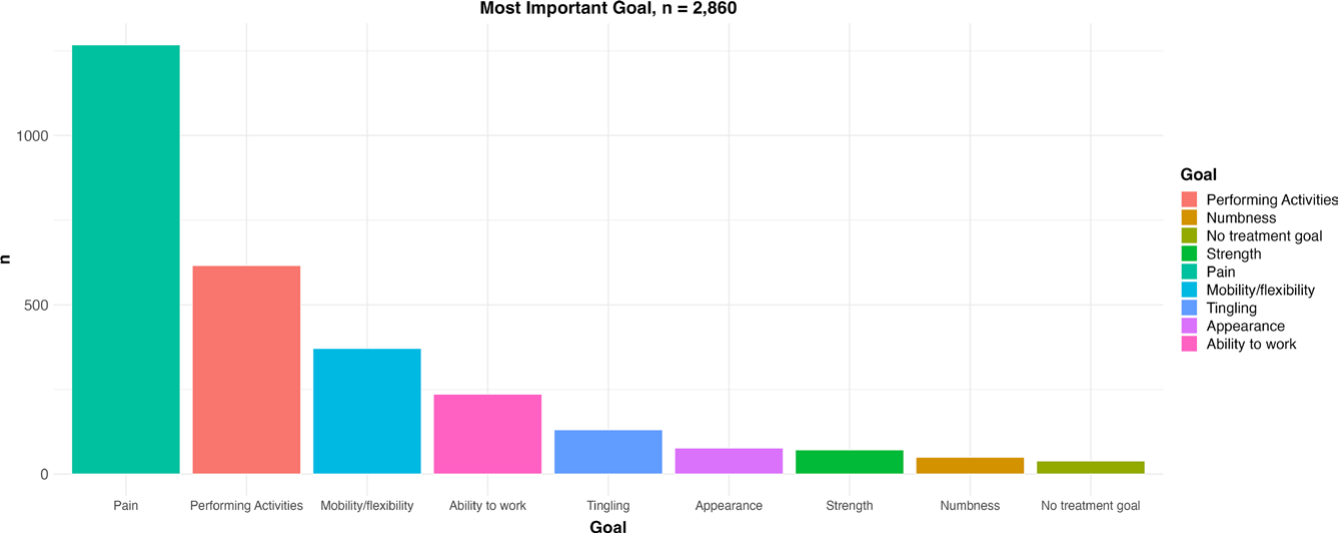
Goal domains chosen as most important in the final version of the PSN.

**Fig. 5.**
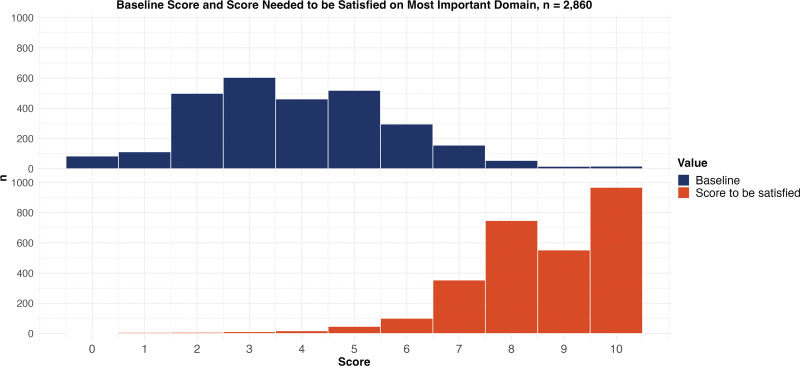
Distribution of scores on the most important goal domain at baseline (*above*) and the score patients reported that they needed to achieve to be satisfied with the treatment results (*below*) in the final version of the PSN. Not all patients want to obtain the maximum score on their most important outcome domain to be satisfied with the treatment results. The median score needed to be satisfied with the treatment result was 9 in the final version. For ease of interpretation, we converted each domain score to the same scale (ie, reversing the scores on the pain, numbness, and tingling domains).

The final PSN was completed by 2860 patients (Table 2). Figure 3 shows the selected information need categories, and Figure 4 shows the distribution of the selected treatment goals. The rating on the most important domain was normally distributed, with a median score of 4 (Fig. 5). The median score needed for the patient to be satisfied with the treatment result was 9 (Fig. 5).

**Table 2.** Baseline Characteristics of Patients Who Completed the Final PSN (*n* = 2860), the Discriminative Validity Sample (*n* = 1985), and Patients Who Participated in the Test-Retest Sample (*n* = 102)

Variable	Sample That Completed the Final PSN (*n* = 2860)	Discriminative Validity Sample (*n* = 1985)	Test-Retest Sample (*n* = 102)
Age, mean (SD), yr	54 (16.3)	59 (13.9)	61 (15.5)
Male sex, no. (%)	1086 (38.0)	704 (35.5)	46 (45.1)
Duration of symptoms, mean (SD), mo	18 (38.2)	17 (33.5)	21 (39.6)
Type of work, no. (%)			
Unemployed due to retirement	695 (24.3)	570 (28.7)	41 (40.2)
Unemployed due to other reason	339 (11.9)	214 (10.8)	6 (5.9)
Light physical labor (eg, office work)	735 (25.7)	468 (23.6)	22 (21.6)
Moderate physical labor (eg, working in a store)	648 (22.7)	438 (22.1)	16 (15.7)
Heavy physical labor (eg, working in construction	443 (15.5)	295 (14.9)	17 (16.7)
Level of education, no. (%)			
None	34 (1.2)	12 (0.6)	1 (1.0)
Primary education (primary school, special primary education)	71 (2.5)	31 (1.6)	1 (1.0)
Primary or prevocational education	323 (11.3)	252 (12.7)	12 (11.8)
Secondary general secondary education	517 (18.1)	356 (17.9)	24 (23.5)
Secondary vocational education and vocational training	599 (20.9)	429 (21.6)	20 (19.6)
Higher general and preuniversity education	251 (8.8)	198 (10.0)	9 (8.8)
Higher vocational education	608 (21.3)	466 (23.5)	21 (20.6)
Scientific education (eg, MSc degree)	299 (10.5)	164 (8.3)	8 (7.8)
Prefer not to say	158 (5.5)	77 (3.9)	6 (5.9)
Body mass index, mean (SD)	26.5 (4.7)	27.2 (4.9)	26.5 (4.4)
Smoking status, no. (%)			
Yes, daily smoker	367 (12.8)	207 (10.4)	10 (9.8)
Yes, passive smoker	15 (0.5)	8 (0.4)	2 (2.0)
Yes, sometimes	140 (4.9)	76 (3.8)	6 (5.9)
No	2338 (81.7)	1694 (85.3)	84 (82.4)
Affected side, no. (%)			
Left	930 (32.5)	607 (30.6)	33 (32.4)
Right	1106 (38.7)	743 (37.4)	40 (39.2)
Both	824 (28.8)	635 (32.0)	29 (28.4)
Hand dominance, no. (%)			
Left	299 (10.5)	199 (10.0)	11 (10.8)
Right	2395 (83.7)	1676 (84.4)	84 (82.4)
Both	166 (5.8)	110 (5.5)	7 (6.9)
Second opinion = no, no. (%)	2475 (86.5)	1781 (89.7)	87 (85.3)
Personal injury lawsuit = no, no. (%)	2801 (97.9)	1960 (98.7)	100 (98.0)

### Discriminative Validity and Test-Retest Reliability

**Fig. 6.**
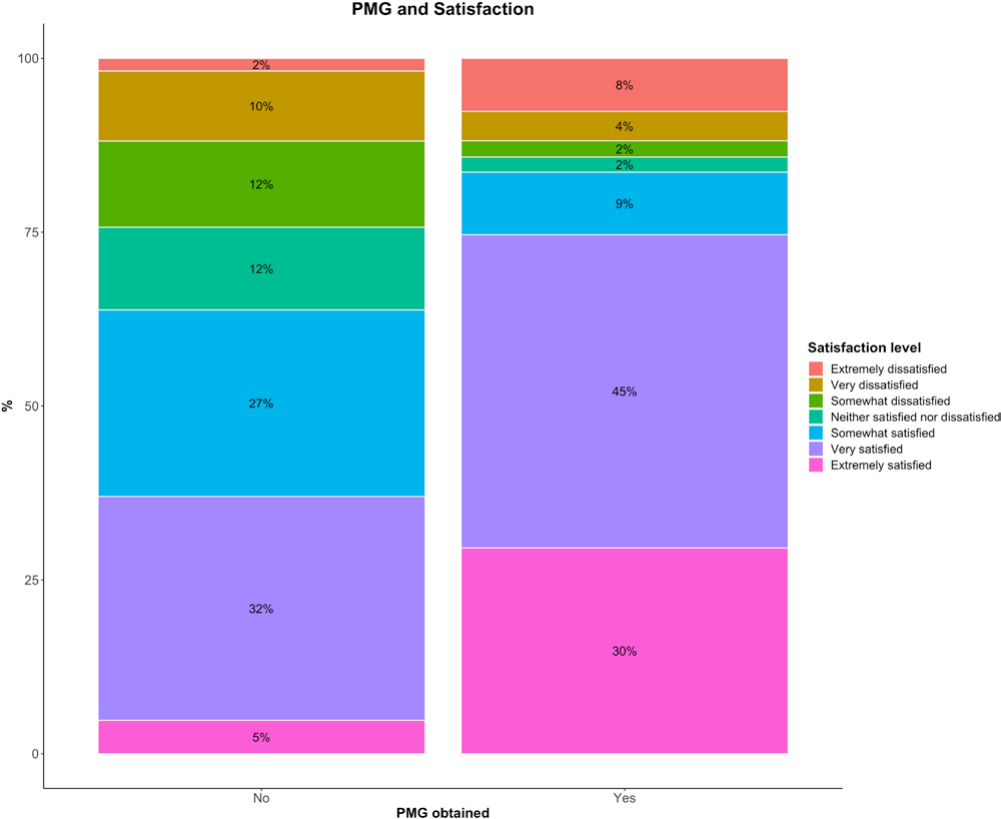
Discriminative validity of the PMG in 1985 patients, demonstrating that patients who obtained their PMG were much more often satisfied with their treatment results compared with patients who did not obtain their PMG, with a medium to large effect size (Cramer V, 0.48; *P* < 0.001).

We included 1985 patients for the discriminative validity analysis (Table 2). Patients who obtained their PMG had better satisfaction with treatment results than those who did not (Fig. 6) (*P* < 0.001). There was a medium to large effect size (Cramer’s V: 0.48), indicating that the PMG has excellent discriminative validity (ie, the ability to distinguish satisfied from dissatisfied patients).

For test-retest reliability, 102 of the 139 invited patients completed both the primary test and the retest within a median interval of 7 days (range, 3 to 11 days). We found small differences between responders and nonresponders in age and type of work. (**See Table, Supplemental Digital Content 4**, which shows nonresponder analysis for the test-retest study, http://links.lww.com/PRS/H16.) There was moderate agreement and reliability for the most important goal domain (Table 3). (**See Table, Supplemental Digital Content 5**, which shows how often the most important goal domain was chosen at the primary test as well as at the retest. The values correspond to the number of patients and the percentage of the row total, except for the “row total” column, where the percentages correspond to the percentage of the column total, http://links.lww.com/PRS/H17.) When the most important goal domain was also chosen as a secondary goal domain in the retest, the test-retest improved to substantial agreement and high reliability (Table 3). (**See Table, Supplemental Digital Content 6**, which demonstrates how often the most important goal domain was chosen at the primary test and also as the most important or as secondary goal domain at the retest. The values correspond to the number of patients and the percentage of the row total, except for the “row total” column, where the percentages correspond to the percentage of the column total, http://links.lww.com/PRS/H18.) We found moderate reliability for the baseline score on the most important goal domain, for the score the patient needed to be satisfied, and for the PMG (Table 3).

**Table 3.** Test-Retest Reliability of the PSN

Test-Retest Variable	Absolute Agreement	Cohen Kappa (95% CI)	ICC (95% CI)	Conclusion
Most important goal domain	58%	0.46 (0.34–0.58)	0.53 (0.38–0.66)	Moderate agreement and reliability
Most important goal domain chosen as most important goal domain or as secondary goal domain at retest	75%	0.68 (0.58–0.79)	0.73 (0.62–0.81)	Substantial agreement and high reliability
Baseline score on most important goal domain	—	—	0.57 (0.42–0.69)	Moderate reliability
Score needed to be satisfied at most important goal domain	—	—	0.64 (0.51–0.74)	Moderate reliability
PMG at most important goal domain	—	—	0.65 (0.53–0.75)	Moderate reliability

ICC, intraclass correlation coefficients.

## DISCUSSION

The PSN focuses on patient-specific information needs and treatment goals, and supports patient-centered care. Although developed in hand and wrist patients, the PSN can be easily modified to unlock its potential for generalization by altering answer options. As part of the PSN, we introduce the PMG as a valid parameter of the improvement an individual wants to obtain in a domain relevant to that individual, given the pretreatment score.

### How to Use the PSN

The PSN can be used as a conversation starter, decision support tool, and expectation management tool during the first consultation. The information needs section helps clinicians to effectively provide information and tailor information provision to the individual patient. For example, knowing a patient’s tendency toward surgery may guide how a clinician proposes noninvasive treatment when more appropriate. The treatment goal aids realistic goal setting, such as if a patient with Dupuytren disease wants to improve his or her hand appearance, but it is unlikely that this will be achieved with treatment. The PMG helps to identify and discuss expectations (eg, if one wants to improve from 2 to 10 to be satisfied, although this may be unrealistic due to comorbidity or symptom duration). The PSN also evaluates treatment success at a personal level.

There was moderate agreement and reliability for the most important goal domain. However, these improved to a substantial agreement and high reliability when also considering agreement if the most important goal domain was also a secondary goal domain in the retest. This indicates that the PSN’s reliability is good enough to identify all patient-relevant goals. Thus, patients find it hard to distinguish between their most important goal and their secondary goal, which may overlap. Our finding that most patients who obtained their PMG were satisfied with their treatment results suggests that their satisfaction was independent of whether their PMG was on their factual primary goal, confirming the PSN’s usability. Clinicians should always consider all goals and not just the most important goal domain.

### Key Considerations

User participation during the development, iterative approach, pilot testing, and mixed-methods study resulted in a content-valid, discriminative, and reliable patient-centered tool. The PSN was easily implemented, and patients deemed it relevant, complete, and understandable. The PSN helps patients prepare for their first consultation, enhances awareness, empowers them to take control of their treatment, and aids shared decision-making. The clinicians indicated that the PSN helps them to identify patients with high or low expectations and respond accordingly. These aspects may improve patients’ experience, expectation management, satisfaction with treatment results, and clinical outcomes.^50^

Compared with existing tools,^32–35^ the PSN adds value. For example, the Canadian Occupational Performance Measure, Goal Attainment Scaling, and Patient-Specific Goalsetting Method tools are completed together with a health care provider. They are relatively time-consuming in clinical practice, and there is a risk of “therapist bias,” as a practitioner may influence these goals. Other tools do not assess patient-specific improvement goals and their relationship with satisfaction with treatment results, whereas the PSN does (ie, the PMG). Furthermore, in contrast with current tools, such as the Patient-Specific Functional Scale, Canadian Occupational Performance Measure, and Patient-Specific Goalsetting Method, the PSN allows distinct International Classification of Functioning, Disability, and Health domains, instead of focusing only on the activities and participation levels. None of the aforementioned tools assesses information needs, but the PSN does. Altogether, the PSN is a unique tool with added value in daily clinic and research.

The distribution of the information need category and goal domain indicates that patients have different needs and goals. This highlights that a personalized treatment strategy, which can be informed by the PSN, is essential. Further, although most people wanted to reach a 9 to be satisfied, many patients consider lower scores satisfactory (ie, not all patients aim for the maximum score). The wide distribution indicates that this is indeed a personalized score, which further adds to the value of the PSN.

The PMG distinguished satisfied patients from dissatisfied patients very well, indicating that it can be used to evaluate the clinical relevance of treatment effects. The PMG is especially beneficial, as it is determined before clinician consultation, providing a proxy for satisfaction with treatment results at a very early stage, presuming what patients think they want is what will satisfy them. Future research may investigate whether the PMG has a greater discriminative capacity for satisfaction than traditional values, such as the minimal important change or the patient acceptable symptom state.

At our sites, a clinician dashboard is used that displays patient characteristics, patient-reported outcome measures, clinician-reported outcomes (eg, goniometry), and prediction models. With the PSN added, health care can be further personalized and data-driven. Nevertheless, the PSN is also valuable as a standalone tool.

We distribute the PSN before surgeon consultation. If treatment is scheduled (eg, surgery or therapy), we allow patients to change previous answers. For example, the patient’s goal may have changed following expectation management during consultation. This strategy is, of course, optional.

### Limitations

Respondents indicate their most important needs and goals without knowing their diagnosis. It may also be difficult for individuals to accurately predict how they will feel about a future score, such as a 9 or 10, since this is an abstract idea that may not match their actual experience when they reach that level. However, focusing on the patient’s most important needs and goals at this early stage benefits clinicians, as they may use these factors in decision-making and expectation management. Although some items may be moving targets (ie, a response shift, as goals may change over time), the PSN discriminated effectively between satisfied and dissatisfied patients. Future research could investigate how needs and goals change over time.

The PSN does not replace traditional outcome measures, and additional time investment should be considered when using it.

Another limitation is the test-retest nonresponse. The small differences between responders and nonresponders seem clinically irrelevant, as age and type of work are unlikely to influence test-retest reliability. Nevertheless, although inevitable in test-retest studies, this may have influenced our findings.

We addressed most issues mentioned by respondents, but we kept the maximum number of information need categories respondents could choose. Obviously, patients have more questions, and clinicians should try to answer them all. However, we considered it essential that, at the least, the most important question is identified and answered, as there is a maximum information load that people can absorb. Therefore, it is essential to see the PSN as a conversation starter. In addition, patients might be better prepared by knowing their most important question.^50^

Another limitation is that we excluded patients with inconsistent answers on the survey. This may have influenced our findings on the understandability of the PSN. However, if we had included these patients, our findings would have been biased; thus, we believe that our decision was the best solution to minimize bias. In addition, although the participants had different educational levels (including lower levels), it remains challenging to reach lower-literacy patients. Future research may specifically target these patients.

Although we performed a crosscultural translation to English, we only tested the Dutch version. Future studies may investigate the PSN in different languages and cultural settings.

## CONCLUSIONS

The PSN is a novel, brief patient-reported tool for identifying individual patient needs and goals. By identifying these needs and goals, clinicians are better equipped to tailor information provision and treatment to the individual patient, enhancing the quality of care. The PSN can help patients to take control of their treatment. It is valid, reliable, and easy to use, especially, but not only, in digital form. The PSN is implementation-ready for hand and wrist care, and can easily be generalized to other fields. The PSN is provided with open access and is free to use.

## DISCLOSURE

Dr. Wouters received funding from ZonMw to support this research. The remaining authors have no conflicting interests in relation to the work presented in this article.

## ACKNOWLEDGMENTS

The authors thank all patients who completed questionnaires as part of their clinical care and agreed that their data could be used anonymously for the present study. In addition, the authors thank the members of the Hand-Wrist Study Group, clinicians, and personnel of Xpert Clinics, Xpert Handtherapie, and Equipe Zorgbedrijven for assisting in the routine outcome measurements that are the basis for this study.

## APPENDIX

The Hand-Wrist Study Group collaborators are as follows: Dirk-Johannes Jacobus Cornelis van der Avoort, MD; Ward Rogier Bijlsma, MD, PhD; Richard ArjenMichiel Blomme, MD; Herman Luitzen de Boer, MD; Gijs Marijn van Couwelaar, MD; Jan Debeij, MD, PhD; Jak Dekker, MSc; Reinier Feitz, MD, PhD; Alexandra Fink, PT; Kennard Harmsen, MD; Lisa Hoogendam, BSc; Steven Eric Ruden Hovius, MD, PhD; Rob van Huis, PT; Richard Koch, MD; Yara Eline van Kooij, PT, MSc; Jaimy Emerentiana Koopman, MD; Alexander Kroeze, MD; Nina Louisa Loos, MSc; Thybout Matthias Moojen, MD, PhD; Mark Johannes Willem van der Oest, PhD; Pierre-Yves Alain Adriaan Pennehouat; PT; Willemijn Anna de Ridder, PT, MSc; Johannes Pieter de Schipper, MD; Karin Schoneveld, PT, MSc; Ruud Willem Selles, PhD; Harm Pieter Slijper, PhD; Jeronimus Maria Smit, MD, PhD; Xander Smit, MD, PhD; John Sebastiaan Souer, MD, PhD; Marloes Hendrina Paulina ter Stege, MSc; Johannes Frederikes Maria Temming, MD; Joris Sebastiaan Teunissen, BSc; Jeroen Hein van Uchelen, MD, PhD; Joris Jan Veltkamp, PT; Guus Maarten Vermeulen, MD, PhD; Erik Taco Walbeehm, MD, PhD; Robbert Maarten Wouters, PT, PhD; Oliver Theodor Zöphel, MD, PhD; and Jelle Michiel Zuidam, MD, PhD.

## Supplementary Material












