# Supplementary material for: Tailoring and Evaluating Treatment with the Patient-Specific Needs Evaluation: A Patient-Centered Approach
Source: Plast Reconstr Surg. 2023 Dec 12;154(4):787–99. doi: 10.1097/PRS.0000000000011199 (PMC11412570; doi:10.1097/PRS.0000000000011199)
Supplement: Supplementary file 1 [file prs-154-0787-s001.pdf]

Main categories	Findings	Quotes
**1: Relevance**	1.1: Most participants found it relevant that their answers could direct the conversation	1.1.1 “I think it's important for a doctor to know that in advance. Know what the situation is” (P1)<br>1.1.2 “easy to give direction to the conversation” (P1)<br>1.1.3 “Time efficient” (P2)
	1.2: Most participants found it relevant that the clinician could respond to expectations	1.2.1 “If you absolutely want to reach a 10, that says something about you” (P1)<br>1.2.2 “it is easier for the doctor to estimate the patient’s expectations” (P3)<br>1.2.3 “I can imagine that it is nice for the practitioner to already know what someone would most like to improve” (P4)
	1.3: Some participants felt well prepared and empowered by filling in the questionnaire	1.3.1 “Yes, also nice for myself to already think about what I would like to know during such a first intake, for example” (P5)<br>1.3.2 “You feel that you are taken seriously” (P6)
**2: Completeness: information need**	2.1 Some participants seem worried about whether the clinician would answer all their questions	2.1.1 FIELD NOTES
	2.2 Many participants deemed it impossible to choose just one answer option	2.2.1 “To me, everything was important” (P2)<br>2.2.2 “asking multiple questions to arrive at an opinion” (P6)
**3: Completeness: treatment goal and improvement goal**	3.1 All participants thought the questions and answer options were complete	3.1.1 ”I believe everyone can find his or her complaint in here” (P7)<br>3.1.2 “Seems all right to me, yes, I can't see one missing” (P1)
**4: Understandability: introductory texts**	4.1 Many participants thought the welcome text was too elaborate	4.1.1 “It could also be said in two sentences” (P7)<br>4.1.2 “Yes, just a good clear explanation” (P6)
	4.2 Some participants missed a sentence on how many options they were allowed to pick at ‘treatment goal’	4.2.1 “It literally says pick one” (P3)<br>4.2.2 “for some people I think it is annoying to have to focus on one thing again” (P3)
**5: Understandability: information need**	5.1 Most participants found the question and answer options clear	5.1.1 ”Give me a second... No, it's clear like this” (P8)
	5.2 Some participants thought they couldn't answer the question without more information beforehand	5.2.1 “You can't really ask about treatment if a diagnosis has not yet been determined” (P3)
**6: Understandability: treatment goal and improvement goal**	6.1 Many participants thought the answer scale for pain was too complicated	6.1.1 “You need to switch the scale” (P3)<br>6.1.2 “With smiley faces for people who think it is complicated” (P6)<br>6.1.3 “I would say 'unbearable pain’ and 'no pain' instead of 'very bad' and 'excellent’” (P3)
	6.2 Some participants found specific words in the domain section too complicated	6.2.1 “Well, I didn't know what pliability was” (P2)
