# Supplementary material for: Tailoring and Evaluating Treatment with the Patient-Specific Needs Evaluation: A Patient-Centered Approach
Source: Plast Reconstr Surg. 2023 Dec 12;154(4):787–99. doi: 10.1097/PRS.0000000000011199 (PMC11412570; doi:10.1097/PRS.0000000000011199)
Supplement: Supplementary file 2 [file prs-154-0787-s002.pdf]

Main categories	Findings	Quotes
**1: Relevance**	1.1 Many clinicians thought the open question for aid and the conversation itself provide enough information to understand what the patients needs are	1.1.1 "you can just have this conversation and then you will get this information too" (C2)<br>1.1.2 "I'm quite satisfied with the information that we already have" (C4)
	1.2 Some clinicians thought the questionnaire might help them to formulate a relevant treatment goal	1.2.1 "I would definitely look it over and see what the patients goal was prior to their visit to the doctor. And of course I would discuss that with them, before coming here, your goal was this, is that still your goal or do you now want something else?" (C1)
	1.3 Many clinicians thought it useful to know what the patients goal is to become satisfied with the treatment results	1.3.1 "If the expectations aren't realistic then I would use it. (…) If it is just regular, then I won't do much with it" (C4)<br>1.3.2 "you can filter out those extremes nicely"(C2)
	1.4 Some clinicians thought it useful that the patient fills in the questionnaire before the first consultation	1.4.1 "I actually think this is a good one, because the patient can tell his own story. So one is a little less likely to be overwhelmed by the opinion of a clinician" (C3)
	1.5 Some clinicians thought it useful to have an image of the patient before the first consultation	1.5.1 "It's interesting, by certain answers, you also get to see a kind of personality" (C5)
**2: Completeness: information need**	2.1 All clinicians think the part on information need is complete	2.1.1 "It's definitely complete, especially the first question" (C1)
**3: Completeness: treatment goal and improvement goal**	3.1 Most clinicians think the part on the treatment goal is complete	3.1.1 "Yes, yes, I think it is fairly complete in terms of complaints" (C3)
**4: Usability for the clinician**	4.1 Barrier: Most clinicians are afraid the questionnaire will cost them more time	4.1.1 "because of course you don't have forever to prepare so I'm not sure I would look at this" (C2)<br>4.1.2 "Example given, 'oh yes, I saw that you are a bricklayer or something' and then you immediately have a conversation and someone also has the feeling that his information is used" (C3)
	4.2 Risk: Some clinicians fear being biased	4.2.1 "Well, I think I have to be very careful not to start with prejudices. Someone has discussed his profession and his complaints, so I'm already starting with a tunnel vision" (C3)<br>4.2.2 "And especially a conversation is dynamic. You can't put at person in a box" (C4)
	4.3 Risk: some clinicians fear that clinicians will only answer the most important answer	4.3.1 "If I look at it quickly, I could just be put on the wrong track when I see that they can only indicate one"<br>4.3.2 "You have to let therapists know that patients are only asked to only choose one answer" (C1)
	4.4 Barrier: Some clinicians thought it hard to use the patients answer to the information need in their consult	4.4.1 "And vice versa, you choose diagnosis and treatment, or advice and future, but that is usually also a multi-question" (C4)<br>4.4.2 "Yeah, I honestly don't know if I'll be using this when they can only choose one option, because then I know okay, they will ask more questions anyway. Do you understand what I mean?" (C2)
**5: Usability/ understandability for patients**	5.1 All clinicians thought the answer scale to pain was too complicated	5.1.1 "If you just use the same scale for everything and not a grade, because now I will be thinking, 'hey, a grade, okay, but I just entered something different; help, did I do that right?'" (C2)
	5.2 Some clinicians think several words and questions are too hard for patients to understand	5.2.1 FIELD NOTE: suggestion to put answers in sentences (narrative mode) (C4, C5, C6)<br>5.2.2 "We have a certain level of intelligence, it's not that I feel elevated, but a majority of patients do not even understand some of the words" (C3)
	5.3 Some clinicians wonder whether the patient answers honestly	5.3.1 "All patients want their doctor to put maximum effort in it" (C5)<br>5.3.2 "But is that realistic?" (C1)
	5.4 Risk: many clinicians are afraid that patients have to answer too many overlapping questions	5.4.1 "Yes, you know, whatever, 'I already filled this in' and then you fill in the question differently than the other one. So then you give a score, you don't look at it carefully, while you might have done that other list very carefully" (C3)
