# Supplementary material for: Tailoring and Evaluating Treatment with the Patient-Specific Needs Evaluation: A Patient-Centered Approach
Source: Plast Reconstr Surg. 2023 Dec 12;154(4):787–99. doi: 10.1097/PRS.0000000000011199 (PMC11412570; doi:10.1097/PRS.0000000000011199)
Supplement: Supplementary file 3 [file prs-154-0787-s003.pdf]

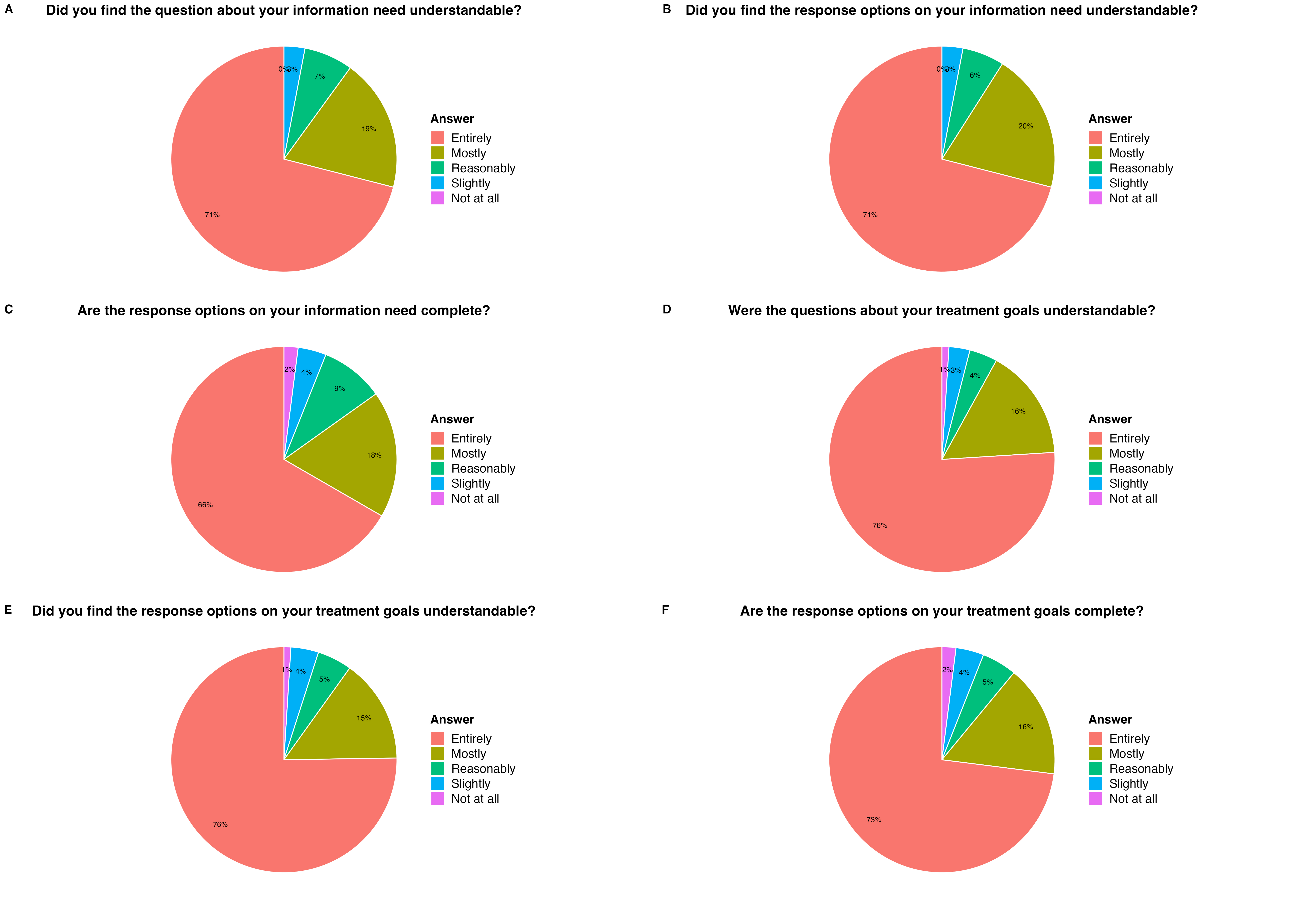
A
Did you find the question about your information need understandable?
0%
3%
7%
19%
71%
Answer
Entirely
Mostly
Reasonably
Slightly
Not at all
B
Did you find the response options on your information need understandable?
0%
3%
6%
20%
71%
Answer
Entirely
Mostly
Reasonably
Slightly
Not at all
C
Are the response options on your information need complete?
2%
4%
9%
18%
66%
Answer
Entirely
Mostly
Reasonably
Slightly
Not at all
D
Were the questions about your treatment goals understandable?
1%
3%
4%
16%
76%
Answer
Entirely
Mostly
Reasonably
Slightly
Not at all
E
Did you find the response options on your treatment goals understandable?
1%
4%
5%
15%
76%
Answer
Entirely
Mostly
Reasonably
Slightly
Not at all
F
Are the response options on your treatment goals complete?
2%
4%
5%
16%
73%
Answer
Entirely
Mostly
Reasonably
Slightly
Not at all
