# Supplementary material for: Tailoring and Evaluating Treatment with the Patient-Specific Needs Evaluation: A Patient-Centered Approach
Source: Plast Reconstr Surg. 2023 Dec 12;154(4):787–99. doi: 10.1097/PRS.0000000000011199 (PMC11412570; doi:10.1097/PRS.0000000000011199)
Supplement: Supplementary file 4 [file prs-154-0787-s004.pdf]

Supplemental Digital Content 4. Non-responder analysis for the test-retest study.

**Variable**	**Non-Responders (n = 37)**	**Responders (n = 102)**	**p-value***	**SMD**
Age, median [IQR]	53.00 [36.00, 63.00]	64.00 [51.25, 73.75]	<0.001	0.691
Sex = male, n (%)	13 (35.1)	46 (45.1)	0.392	0.204
Duration of symptoms in months, median [IQR]	12.00 [6.00, 28.00]	11.00 [5.00, 18.75]	0.171	0.305
Type of work, n (%)			0.009	0.745
Unemployed due retirement	6 (16.2)	41 (40.2)		
Unemployed due other reason	4 (10.8)	6 (5.9)		
Light physical labor (e.g., office work)	10 (27.0)	22 (21.6)		
Moderate physical labor (e.g., working in a store)	14 (37.8)	16 (15.7)		
Heavy physical labor (e.g., working in construction	3 (8.1)	17 (16.7)		
Level of education (%)			0.950	0.328
None	1 (2.7)	1 (1.0)		
Primary education (primary school, special primary education)	0 (0.0)	1 (1.0)		
Primary or pre-vocational education (such as (in Dutch) LTS, LEAO, LHNO, Huishoudschool, VMBO)	4 (10.8)	12 (11.8)		
Secondary general secondary education (such as (in Dutch) MAVO, (M)ULO, MBO-short, VMBO-t)	6 (16.2)	24 (23.5)		

Secondary vocational education and vocational training (such as (in Dutch) MKBO-long, MTS, MEAO, BOL, BBL, INAS)	8 (21.6)	20 (19.6)		
Higher general and pre-university education (such as (in Dutch) HAVO, VWO, Atheneum, Gymnasium, HBS, MMS)	2 (5.4)	9 (8.8)		
Higher vocational education (such as (in Dutch) HBO, HTS, HEAO, HBO-V, university graduates	9 (24.3)	21 (20.6)		
Scientific education (e.g., MSc.)	4 (10.8)	8 (7.8)		
Prefer not to say	3 (8.1)	6 (5.9)		
Body Mass Index, median [IQR]	26.00 [23.00, 28.00]	26.00 [23.00, 29.00]	0.617	0.042
Smoking status, n (%)			0.366	0.356
Yes, daily smoker	7 (18.9)	10 (9.8)		
Yes, passive smoker	0 (0.0)	2 (2.0)		
Yes, sometimes	1 (2.7)	6 (5.9)		
No	29 (78.4)	84 (82.4)		
Affected side, n (%)			0.953	0.059
Left	13 (35.1)	33 (32.4)		
Right	14 (37.8)	40 (39.2)		
Both	10 (27.0)	29 (28.4)		
Dominance, n (%)			0.560	0.223

Left	3 (8.1)	11 (10.8)		
Right	33 (89.2)	84 (82.4)		
Both	1 (2.7)	7 (6.9)		
Second opinion = no, n (%)	34 (91.9)	87 (85.3)	0.460	0.209
Personal injury lawsuit = no, n (%)	36 (97.3)	100 (98.0)	1.000	0.049

*Continuous variables were compared using the Kruskal-Wallis Rank Sum Test and dichotomous or categorical variables using a Chi-Square test.
