# Supplementary material for: Tailoring and Evaluating Treatment with the Patient-Specific Needs Evaluation: A Patient-Centered Approach
Source: Plast Reconstr Surg. 2023 Dec 12;154(4):787–99. doi: 10.1097/PRS.0000000000011199 (PMC11412570; doi:10.1097/PRS.0000000000011199)
Supplement: Supplementary file 5 [file prs-154-0787-s005.pdf]

Supplemental Digital Content 5. Cross table demonstrating how often the most important goal domain was chosen at the primary test as well as at the retest. The values correspond to the number of patients and the percentage of the row total, except for the "Row total" column, where the percentages correspond to the percentage of the column total.

	**Most important goal domain at retest**								
**Most important goal domain at primary test**	*Pain*	*Activities*	*Flexibility/ Mobility*	*Work*	*Tingling*	*Strength*	*Appearance*	*Numbness/ Sensation*	*Row total*
Pain	24 (69%)	5 (0,14%)	3 (9%)	2 (6%)	0 (0%)	0 (0%)	1 (3%)	0 (0%)	35 (34%)
Activities	7 (29%)	15 (63%)	0 (0%)	1 (4%)	0 (0%)	0 (0%)	0 (0%)	1 (4%)	24 (24%)
Flexibility/Mobility	2 (13%)	3 (20%)	8 (53%)	0 (0%)	0 (0%)	0 (0%)	2 (13%)	0 (0%)	15 (15%)
Work	1 (13%)	2 (25%)	1 (13%)	3 (38%)	0 (0%)	0 (0%)	0 (0%)	1 (13%)	8 (8%)
Tingling	0 (0%)	1 (17%)	0 (0%)	0 (0%)	5 (8%)	0 (0%)	0 (0%)	0 (0%)	6 (6%)
Strength	2 (33%)	2 (33%)	0 (0%)	0 (0%)	0 (0%)	1 (0,167)	1 (17%)	0 (0%)	6 (6%)
Appearance	0 (0%)	0 (0%)	1 (50%)	0 (0%)	0 (0%)	0 (0%)	1 (50%)	0 (0%)	2 (2%)
Numbness/Sensation	0 (0%)	1 (20%)	1 (20%)	0 (0%)	1 (20%)	0 (0%)	0 (0%)	2 (40%)	5 (5%)
No treatment goal	0 (0%)	0 (0%)	0 (0%)	0 (0%)	1 (100%)	0 (0%)	0 (0%)	0 (0%)	1 (1%)
Column total	36 (35%)	29 (28%)	14 (14%)	6 (6%)	7 (7%)	1 (1%)	5 (5%)	4 (4%)	102 (100%)
