# Supplementary material for: Tailoring and Evaluating Treatment with the Patient-Specific Needs Evaluation: A Patient-Centered Approach
Source: Plast Reconstr Surg. 2023 Dec 12;154(4):787–99. doi: 10.1097/PRS.0000000000011199 (PMC11412570; doi:10.1097/PRS.0000000000011199)
Supplement: Supplementary file 6 [file prs-154-0787-s006.pdf]

**Supplemental Digital Content 6.** This cross table demonstrates how often the most important goal domain was chosen at the primary test and also as the most important or as secondary goal domain at the retest. The values correspond to the number of patients and the percentage of the row total, except for the "Row total" column, where the percentages correspond to the percentage of the column total.

| | **Most important or secondary goal domain chosen at the retest** | | | | | | | | |
|---|---|---|---|---|---|---|---|---|---|
| **Most important goal domain chosen at primary test** | *Pain* | *Activities* | *Flexibility/ Mobility* | *Work* | *Tingling* | *Strength* | *Appearance* | *Numbness/ Sensation* | *Row total* |
| *Pain* | 31 (89%) | 2 (6%) | 1 (3%) | 0 (0%) | 0 (0%) | 0 (0%) | 1 (3%) | 0 (0%) | 35 (34%) |
| *Activities* | 5 (21%) | 19 (79%) | 0 (0%) | 0 (0%) | 0 (0%) | 0 (0%) | 0 (0%) | 0 (0%) | 24 (24%) |
| *Flexibility/Mobility* | 1 (7%) | 3 (20%) | 9 (60%) | 0 (0%) | 0 (0%) | 0 (0%) | 2 (13%) | 0 (0%) | 15 (15%) |
| *Work* | 1 (13%) | 1 (13%) | 0 (0%) | 5 (63%) | 0 (0%) | 0 (0%) | 0 (0%) | 1 (13%) | 8 (8%) |
| *Tingling* | 0 (0%) | 1 (17%) | 0 (0%) | 0 (0%) | 5 (80%) | 0 (0%) | 0 (0%) | 0 (0%) | 6 (6%) |
| *Strength* | 2 (33%) | 1 (17%) | 0 (0%) | 0 (0%) | 0 (0%) | 2 (33%) | 1 (17%) | 0 (0%) | 6 (6%) |
| *Appearance* | 0 (0%) | 0 (0%) | 0 (0%) | 0 (0%) | 0 (0%) | 0 (0%) | 2 (100%) | 0 (0%) | 2 (2%) |
| *Numbness/Sensation* | 0 (0%) | 1 (20%) | 0 (0%) | 0 (0%) | 0 (0%) | 0 (0%) | 0 (0%) | 4 (80%) | 5 (5%) |
| *No treatment goal* | 0 (0%) | 0 (0%) | 0 (0%) | 0 (0%) | 1 (100%) | 0 (0%) | 0 (0%) | 0 (0%) | 1 (1%) |
| *Column total* | 40 (39%) | 28 (28%) | 10 (10%) | 5 (5%) | 6 (6%) | 2 (2%) | 6 (6%) | 5 (5%) | 102 (100%) |
